# A Case of Retained Viable Appendiceal Segment after Perforated Appendicitis

**DOI:** 10.70352/scrj.cr.26-0418

**Published:** 2026-08-04

**Authors:** Masato Hayashi, Masanori Kotake, Hiroki Kitabayashi, Kazuki Kato, Kazuyoshi Mitta, Daisuke Fujimori, Ryosuke Gabata, Koichiro Sawada, Kaeko Oyama, Takuo Hara, Kazuhiro Nomoto, Noriyuki Inaki

**Affiliations:** 1 Department of Surgery, Koseiren Takaoka Hospital, Takaoka, Toyama, Japan; 2 Department of Pathology, Koseiren Takaoka Hospital, Takaoka, Toyama, Japan; 3 Department of Gastrointestinal Surgery/Breast Surgery, Kanazawa University Graduate School of Medical Sciences, Kanazawa, Ishikawa, Japan

**Keywords:** appendiceal autoamputation, interval appendectomy, perforated appendicitis

## Abstract

**INTRODUCTION:**

Conservative management of perforated appendicitis is widely accepted; however, residual appendiceal tissue may persist and lead to delayed complications. We report a rare case of a retained viable appendiceal segment identified during interval appendectomy (IA).

**CASE PRESENTATION:**

An 82-year-old patient was treated conservatively for perforated appendicitis without abscess formation but with marked periappendiceal inflammatory changes. Two months after the initial treatment, an IA was planned. Follow-up CT demonstrated enlargement of the appendix compared with the initial imaging. Intraoperatively, the appendix was found to be transected at the mid-portion. The distal segment was separated from the proximal stump and encapsulated by the omentum and small intestine with dense adhesions. This distal segment remained viable because it retained blood supply through the mesoappendix and showed inflammatory enlargement. Histopathological examination demonstrated preserved appendiceal wall structure with inflammatory cell infiltration, without evidence of necrosis or malignancy. The postoperative course was uneventful, and the patient was discharged on POD 3.

**CONCLUSIONS:**

A retained viable appendiceal segment may persist and enlarge after perforated appendicitis treated conservatively, potentially leading to delayed complications such as secondary perforation or abscess formation; therefore, surgeons should be aware of this possibility and consider IA in selected patients. Careful attention should be paid to ensure complete removal of the appendix, including any detached distal segments.

## Abbreviations

CRP
: C-reactive protein level
HE
: hematoxylin eosin
IA
: interval appendectomy
WBC
: white blood cell count

## INTRODUCTION

Conservative management with antibiotics is a widely accepted strategy for perforated appendicitis, particularly in cases accompanied by abscess formation. However, residual appendiceal tissue may persist after initial treatment, potentially resulting in recurrent inflammation or delayed complications. Although autoamputation of the appendix has been rarely reported, the presence of a viable retained appendiceal segment is extremely uncommon. We report a case of a retained viable appendiceal segment identified during IA following conservative treatment for perforated appendicitis.

## CASE PRESENTATION

**Fig. 1:**
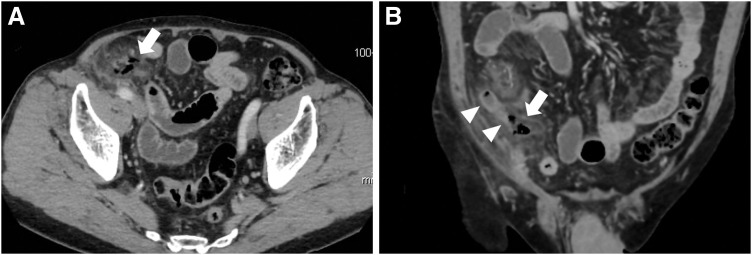
Initial CT findings. (**A**, **B**) Contrast-enhanced CT at initial presentation showing perforation of the appendix at the mid-portion with adjacent free air (arrow). Marked periappendiceal fat stranding is visible, though the contour of the appendix remains largely delineable (arrowheads).

An 82-year-old patient presented with abdominal pain and was diagnosed with perforated appendicitis. Initial CT showed no abscess formation but revealed marked periappendiceal fat stranding (**Fig. 1A** and **1B**). Laboratory findings on admission showed an elevated WBC count of 14900/μL and a CRP level of 9.5 mg/dL. The patient was managed conservatively with antibiotics, leading to clinical improvement.

**Fig. 2:**
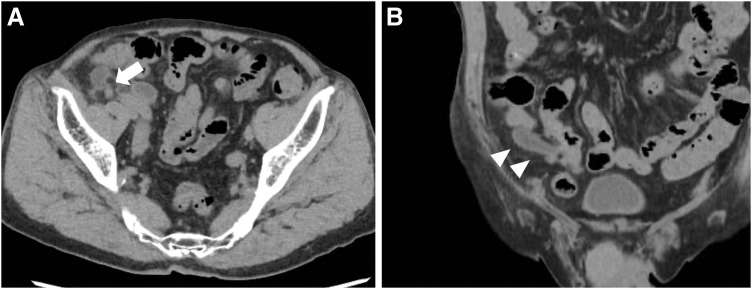
Follow-up CT before IA. (**A**, **B**) Follow-up CT showing resolution of the acute periappendiceal inflammation. Discontinuity between the appendiceal base and the distal segment (arrow) suggests transection. The distal segment appears adherent to the adjacent small intestine, and the distal tip is enlarged (arrowhead). IA, interval appendectomy

Two months later, an IA was scheduled. Prior to surgery, laboratory findings had normalized (WBC 5100/μL, CRP 0.15 mg/dL). Total colonoscopy revealed no abnormalities at the appendiceal orifice. However, follow-up CT demonstrated enlargement of the appendix compared with the initial imaging (**Fig. 2A** and **2B**). Although appendiceal transection was not suspected preoperatively, retrospective review after surgery demonstrated subtle discontinuity between the appendiceal base and the enlarged distal segment.

**Fig. 3:**
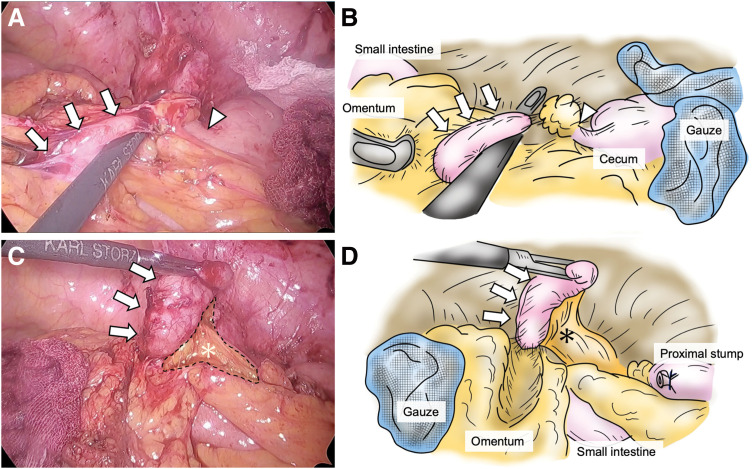
Intraoperative findings. (**A**, **B**) Intraoperative view showing the appendix transected at the mid-portion. The proximal stump is identified at the cecum (arrowhead), while the distal segment (arrows) is encapsulated by the omentum and small intestine with dense adhesions. (**C**, **D**) The mesoappendix supplying the distal segment is preserved (asterisk), suggesting maintained blood flow.

During the IA, the appendix was found to be transected at the mid-portion. The proximal appendiceal segment appeared considerably smaller than expected based on the preoperative CT findings, prompting further exploration. Further dissection revealed that the distal appendiceal segment had completely separated and was embedded within dense adhesions involving the omentum and small intestine (**Fig. 3A** and **3B**). This distal segment was encapsulated and firmly adherent to the surrounding tissues. Notably, the segment remained viable because it retained blood supply through the mesoappendix (**Fig. 3C** and **3D**). The segment appeared enlarged and inflamed. Complete resection of the appendix, including the detached distal segment, was performed successfully.

**Fig. 4:**
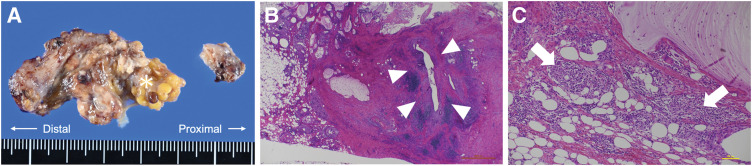
Macroscopic and histopathological findings. (**A**) Macroscopic view of the resected specimen showing the separated distal appendiceal segment connected to the mesoappendix (asterisk), confirming a preserved vascular supply. (**B**) Histopathological examination showing preserved appendiceal wall architecture (arrowheads) with inflammatory cell infiltration (HE stain, ×20), with no evidence of necrosis or malignancy. (**C**) Higher magnification (HE stain, ×100) demonstrating inflammatory cell infiltration extending beyond the appendiceal wall into the adipose tissue of the mesoappendix (arrows). HE, hematoxylin eosin

Histopathological examination revealed a preserved appendiceal wall architecture with inflammatory cell infiltration; no evidence of necrosis or malignancy was observed (**Fig. 4A**–**4C**). The postoperative course was uneventful, and the patient was discharged on POD 3.

## DISCUSSION

In the present case, the appendix was transected at the mid-portion following perforated appendicitis, leaving the distal segment within the abdominal cavity. This segment was encapsulated by the omentum and small intestine, forming a localized inflammatory mass. Crucially, the retained segment remained viable due to a preserved blood supply through the mesoappendix, as confirmed both intraoperatively and histopathologically.

Autoamputation of the appendix is an extremely rare phenomenon, generally resulting from severe inflammation that leads to ischemia, necrosis, and subsequent separation of the tissue.^1)^ Most previously reported cases involve necrotic or nonviable appendiceal fragments, often identified within abscess cavities or as free intraperitoneal bodies.^2–4)^ In contrast, cases in which the amputated segment remains viable are exceptionally rare.

A recent report by Wang et al. described a case of appendiceal autoamputation in which the distal segment remained viable due to preserved blood supply through the mesoappendix—a condition referred to as “pseudo-duplication of the appendix.”^5)^ Additionally, recent reports have associated appendiceal autoamputation with neoplastic conditions or congenital anomalies.^5,6)^ These findings suggest that a preserved vascular supply can prevent necrosis even after appendiceal separation. However, cases in which a viable retained segment arises purely from inflammatory processes are extremely rare. Previous studies have suggested that such retained tissue can lead to chronic inflammation, fistula formation, or other complications if left untreated.^3,7,8)^

In our case, the retained distal segment showed preserved wall architecture and inflammatory infiltration without necrosis, confirming its viability. Following transection, the segment likely lost its normal drainage pathway, leading to luminal obstruction, progressive distension, and persistent inflammation. The follow-up CT prior to surgery showed enlargement of the segment compared to the initial presentation, suggesting ongoing pathological progression. An important lesson from this case is the value of careful preoperative assessment of follow-up CT before IA. Although appendiceal transection was not recognized prospectively in our patient, retrospective review demonstrated subtle discontinuity between the appendiceal base and the enlarged distal segment. Awareness of these imaging findings may alert surgeons to the possibility of a retained viable appendiceal segment and facilitate more meticulous intraoperative exploration. Had surgical intervention been further delayed, the patient might have faced secondary perforation, generalized peritonitis, or recurrent abscess formation. Notably, this case did not involve an initial abscess but rather marked periappendiceal inflammation. This indicates that even in cases without a primary abscess, a retained appendiceal segment can persist and remain clinically significant.

Another important intraoperative lesson is to correlate the operative findings with the preoperative CT. In the present case, the proximal appendiceal segment identified around the cecum appeared considerably smaller than expected based on the preoperative CT findings. This discrepancy prompted further exploration, leading to identification of the retained distal segment. During IA in patients with severe inflammation or dense adhesions, surgeons should confirm that the appendix identified intraoperatively corresponds to the appendix anticipated on preoperative CT. If a discrepancy is noted, such as an unexpectedly short appendiceal stump, further exploration should be performed to identify a possible retained distal segment. Careful confirmation of appendiceal continuity, whether from the base to the tip or from the tip to the base, may help prevent incomplete resection and avoid leaving behind viable residual tissue.

From a surgical perspective, complete appendiceal removal is essential. Residual appendiceal tissue is a known cause of recurrent inflammation, such as stump appendicitis.^8)^ Both emergency and interval appendectomies require careful identification and resection of the entire appendix, including the distal tip. In cases of severe inflammation, the appendix may be fragmented or partially transected, significantly increasing the risk of leaving viable residual tissue behind.

The indication for routine IA after successful conservative management remains controversial. However, the present case suggests that IA may be particularly beneficial in selected patients with suspicious follow-up imaging findings, such as appendiceal enlargement or possible discontinuity, because retained viable appendiceal tissue may progress to delayed perforation or abscess formation if left untreated.

In summary, this case highlights 3 critical clinical points: (1) retained appendiceal segments can persist and enlarge over time even without initial abscess formation; (2) complete resection is essential to prevent residual disease; and (3) even a detached appendiceal segment may remain viable and clinically relevant if its blood supply is preserved.

## CONCLUSIONS

A retained viable appendiceal segment may persist and enlarge after perforated appendicitis treated conservatively and may lead to delayed complications such as perforation or abscess formation. Surgeons should be aware of this possibility and consider IA in selected patients. Careful preoperative evaluation of follow-up CT and meticulous intraoperative confirmation of the entire appendix are essential to avoid leaving behind viable residual appendiceal tissue.

## DECLARATIONS

### Availability of data and materials

Data sharing is not applicable to this article because datasets were neither generated nor analyzed for the case report.
